# Shape-modification of patterned nanoparticles by an ion beam treatment

**DOI:** 10.1038/srep08523

**Published:** 2015-02-17

**Authors:** Kyong Chan Heo, Jin Seog Gwag

**Affiliations:** 1 Department of Physics Yeungnam University, 214-1 Dae-dong, Gyeongsan 712-749, Korea

## Abstract

This paper evaluated a practical approach to the fabrication of arrays of non-spherical nanoparticles by colloidal etching without a mask involving exposure to a low energy ion beam. A spherical nanoparticle array was transferred using a soft nanolithography technique, which is a simple and effective pattern transfer method for nanostructures on the surface of thin adhesive polymers on a planar substrate, after placing the spherical nanoparticles on a patterned PDMS [poly(dimethysiloxane)] stamp produced from a patterned Si wafer. The resulting non-spherical nanoparticle array was driven from a spherical nanoparticle array shape-modified by ion beam irradiation. A well-arrayed layer of cone-like-shapes were produced using a head-on ion beam for different exposure times. Also, a variety of mushroom-like-shapes depending on the exposure angle were produced on a substrate with a well-arranged spherical nanoparticle array. This technique has potential applications in nanophotonics, field emission displays (FEDs) and microfluid.

The assembly of colloidal nanoparticles into non-close-packed (ncp) arrays on substrates has attracted considerable interest for potential applications in optoelectronics, photonics, storage devices, and chemical and bio-sensors^1^,^2^,^3^,^4^. Many potential applications of nanoparticle arrays in nanotechnology require a virtually defect-free array over a large area. In addition, controlling the particle shape, size, and surface structure is important^5^,^6^,^7^ owing to their special structural characteristics and optical properties. Furthermore, a precise array of nano- and micro scaled functional particles at a desired position on a substrate is essential for promising device applications. A range of manipulation techniques, such as optical tweezers, microrobots, electron beam lithography, laser beam, and photolithography have been shown to accurately arrange nanoparticles at a desired position on a substrate^8^,^9^,^10^,^11^. Recently, the fabrication of metallic nanoparticles over a large substrate area has attracted considerable attention for a variety of applications, such as plasmonic nanolithography, bio/medicine sensors and imprinting lithography^12^,^13^,^14^,^15^,^16^,^17^,^18^. For example, sharp metal tips, plasmonic lenses, nanopins, and sharp metallic nanowedges and grooves have been researched extensively because of their special surface structures and optical features^11^,^12^,^13^,^14^,^15^,^16^,^17^. On the other hand, these methods are expensive, complex and have low throughput. In addition, a conventional fabrication methods using nanolithography including reactive ion etching (RIE) with a photo resist are also complicated and expensive producing well-ordered metallic nanostructures over a large surface area. Therefore, a rubbing technique producing an array of colloidal particles by dry manual assembly on patterned substrates has been proposed to apply it to larger substrates^16^. The form of nanoparticles used in these research fields has been restricted to the spherical type, which can be symbolic as the representative shape of building blocks arrayed well into the patterned nanostructure to transfer particles well on patterned substrates. A spherical shape, however, is impractical in most applications requiring advanced technologies with innovative ideas or concepts. Nanoparticle arrays with a range of shapes can widen the application fields and expand the creative functionalities.

This paper present a technique producing non-spherical nanoparticle arrays by colloidal etching without a mask through low energy ion beam exposure. A well-aligned layer of cone-like-shapes were fabricated by frontal ion beam exposure at different exposure times. In addition, a variety of mushroom-like-shapes depending on the exposure angle were fabricated on substrates with a well-arranged spherical nanoparticle array. In this experiment, a spherical nanoparticle array was generated using a soft nanolithography technique, which is a simple and effective pattern transfer method for nanostructures on a plane substrate after placing spherical nanoparticles on a patterned PDMS [poly(dimethysiloxane)] stamp produced from a patterned Si wafer using a rubbing technique. The size and shape of the nanoparticle transferred to the substrate were also modified by exposure to an ion beam. The proposed methods provide a fast and simple method for fabricating reproducible and controllable patterned nanoparticle arrays.

## Results

**Figure 1:**
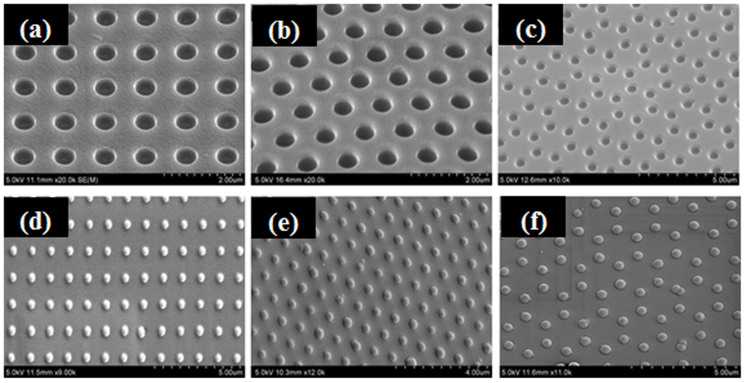
SEM images of various patterned nanowell arrays on a PDMS stamp fabricated by patterned Si-wafers using this soft-lithography technique.

Using the soft-lithography-method, a large-scale monolayer nanoparticle array was fabricated on a glass substrate. Figure 1a-c shows SEM images of various patterned nanowell arrays (a: square type, b: rhombus type, c: hexagonal type) on a PDMS stamp molded by patterned Si-wafers using a soft-lithography method (Supporting information Figure S1). When rubbing silica particles with a 600 nm diameter on the patterned nanowell of a PDMS stamp based on the soft lithography method, each silica particle positioned itself precisely on the each patterned nanowell on the PDMS stamps, as shown in Fig. 1d-f. The center-to-center distance between the nearest 600 nm diameter silica particles was approximately 1.1 μm. The result showed a large area of well-ordered 2D nanoparticle arrays patterned on a planer substrate. In addition, this method can be used to obtain patterned nanoparticle arrays without aggregation.

**Figure 2:**
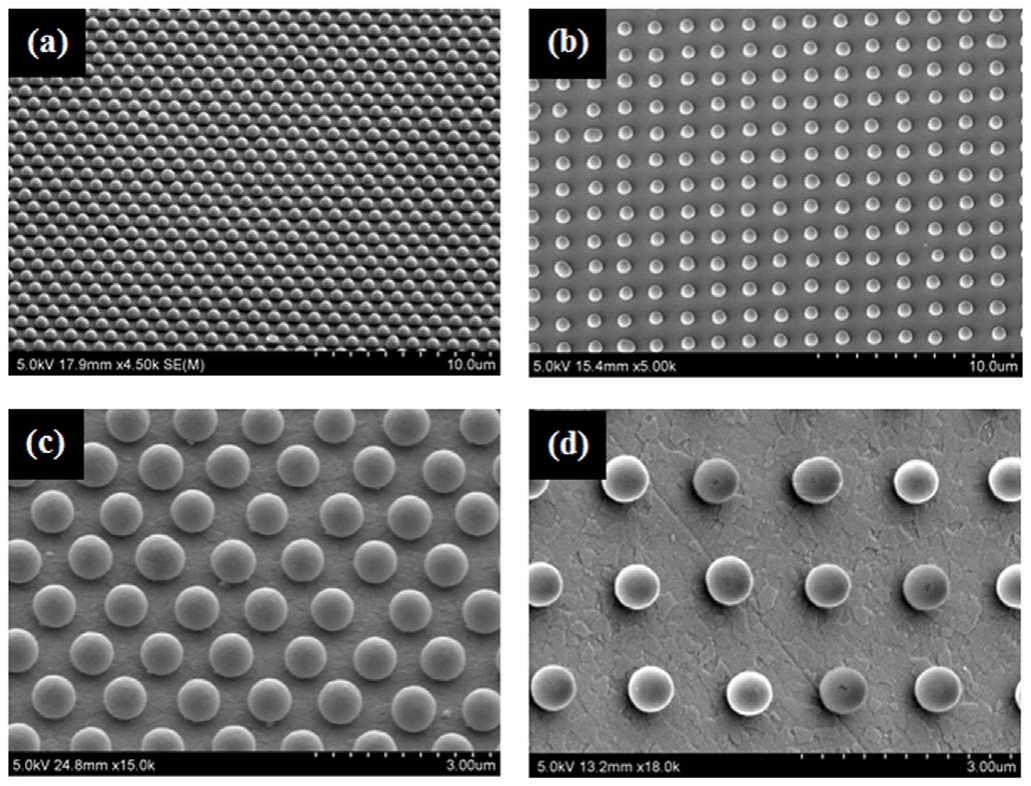
(a-b) Square and hexagonal arrays of nano-bead transferred into the surface of a glass substrate that was pin-coated with NOA60, respectively. (c-d) SEM images of the morphology of silica particles arranged in the square and hexagonal annealed in air at 500°C for 2 h to remove the NOA60 polymer film on the substrate, respectively.

Figure 2a-b shows two pattern arrays of nanoparticles transferred to the surface of a glass substrate that had been spin-coated with NOA60 polymer using this technique. The results confirmed good transfer of a patterned array of nanoparticles from a PDMS surface to the glass substrate (Supporting information Figure 3S). The successful transfer suggests that the interaction between nanoparticles and the PDMS surface was weaker than that between the nanoparticles and the NOA60 polymer film on a substrate when the sample was cured with UV light for more than 20 min. To minimize the effect on the physical and chemical properties of the NOA60 polymer as the molecular glue coated on substrate with the functional nanoparticle arrays for promising applications, this study examined the structure and thermal stability of the patterned nanoparticle arrays annealed above 500°C to remove the NOA60 polymer film from the substrate, which can act as a contaminant. The residue was also removed during ion beam exposure, but the polymer film positioned on the contact region between the nanoparticle and the substrate was not removed due to a screen effect and still as a glue to maintain the position order even under coarse processing. Figure 2c-d shows SEM images of the morphology of the samples annealed in air at 500°C for 2 h to remove the NOA60 polymer film on the substrate. The ordered nanoparticles on the substrate were stable without any aggregation of the patterned nanoparticle array up to 550°C. Although there are point defects (roughly ten particles in the 100 × 80 μm^2^) in patterned nanoparticle array layer transferred onto a glass substrate, uniformly ordered nanoparticle layer of average 600 nm silica particle measured at larger 100 × 80 μm^2^ are obtained by using this method (Supporting information Figure S4).

To determine the modification of the shape and size of the nanoparticles, this study examined the surface morphology of patterned nanoparticles transferred to a glass substrate as a function of the ion beam exposure time and exposure angle with an ion beam energy of 600 eV (Supporting information Figure S3).

**Figure 3:**
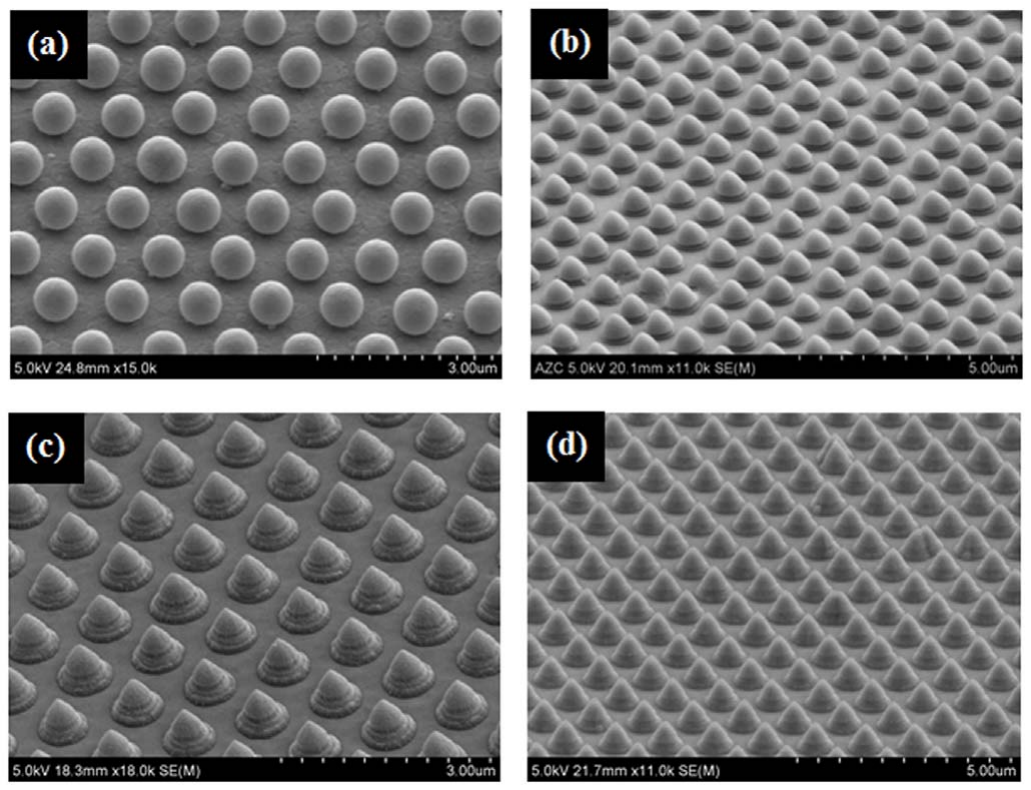
Characteristics of the morphology of the patterned nanoparticle arrays according to the ion beam exposure conditions. (a) under 0 eV, (b) under 600 eV for 2 min and 30 s, (c) at 600 eV for 4 min and 30 s, and (d) SEM image of Al-deposited patterned colloidal silica particle array on the substrate, which was prepared under the ion beam exposure conditions shown in Fig. 3c.

Figure 3 presents SEM images showing several features of the morphology of patterned nanoparticle arrays before and after ion beam exposure. Figure 3(a) shows SEM image of the morphology of the samples annealed in air at 500°C for 2 h to remove the NOA60 polymer film from the substrate before ion beam irradiation. As shown in Fig. 3b-c, cone-like-shaped nanoparticles were generated from the spherical form at ion beam energy of 600 eV normal to the surface direction and the shape was sharper according to the ion beam exposure time. These results show that it is possible to control the shape and size without aggregation or dissipation of the patterned nanoparticle arrays on a glass substrate by ion beam exposure. An Al thin film was deposited on the sample shown in Fig. 3c by sputtering to determine if the transferred array has the required position-stability under high vacuum processes, such as sputtering, to form the surfaces of the colloidal nanoparticles enclosed in the metal thin film shell. RF magnetron sputtering was performed on the patterned silica nanoparticle array on a substrate at room temperature for 10 minutes at 40 W. Figure 3d presents SEM images of Al-deposited patterned nano silica particles array. As expected, the array built up by soft lithography stands rigidly with firm position-stability without being blown away or dissipated under the high vacuum (Supporting information Figure S5).

**Figure 4:**
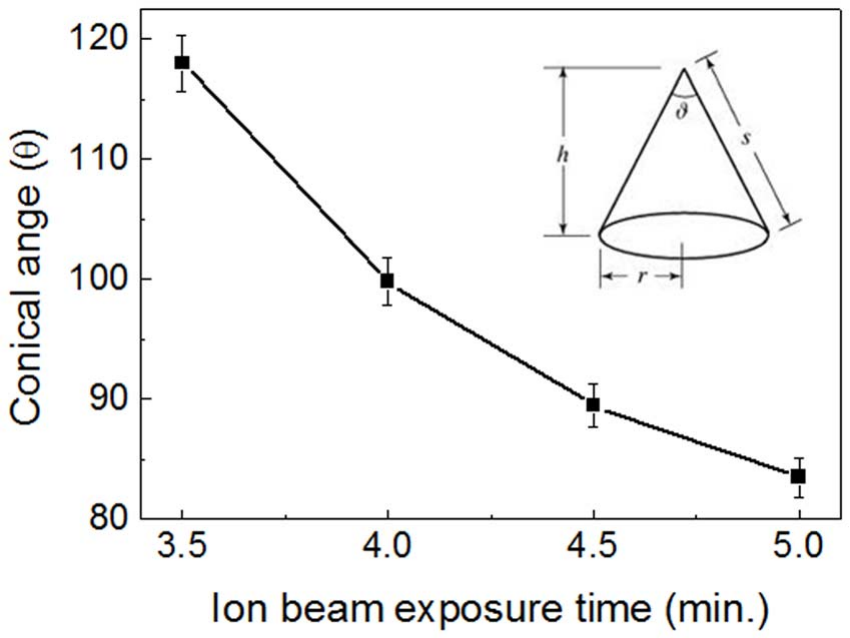
Variations of conical angle depending on the ion beam exposure time.

Figure 4 shows the changes in the conical angle according to the ion beam exposure time. As a result, depending on the application, the shape of the cone is controlled by the exposure time.

**Figure 5:**
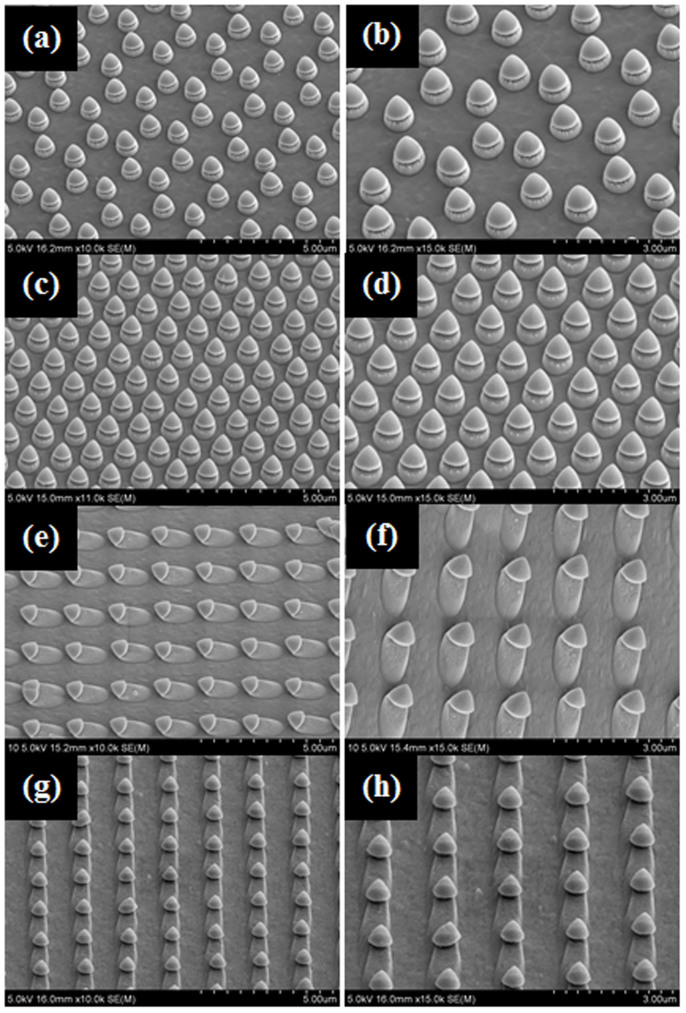
SEM images showing nonspherical array forms modified from the spherical nanoparticle array according to the exposure angles of the ion beam when ion beam energy, beam current density, and exposure times were fitted with 600 eV and 500 μA/cm^2^, and 4 min and 30 s, respectively. (a) and (b) (zoomed image) under an exposure angle of 20°, (c) and (d) (zoomed image) under an exposure angle of 30°, (e) and (f) (zoomed image) under exposure angle of 60°, and (g) and (h) (zoomed image) under an exposure angle of 70°.

At this time, the exposure angle of the ion beam was adjusted to generate another non-spherical nanoparticle array. When the spherical nanoparticle array on the substrate tilted to one direction was irradiated with the ion beam, it was modified to another non-spherical nanoparticle array, a mushroom-like-shape, as shown in Fig. 5. Figure 5 presents SEM images of the non-spherical array formed by modifying the spherical nanoparticle array according to the exposure angles of the ion beam. Figure 5 (a) and (b) show SEM images of the substrate after ion beam exposure when the exposure angle of the ion beam was 20° with respect to the normal direction of the substrate with a hexagonal type nanoparticle patterned array. A uniform and slightly tilted mushroom-like-shape was observed. Figure 5 (c) and (d) present SEM images when the exposure angle of the ion beam was 30° with respect to the normal direction of the substrate showing a lozenge-shaped nanoparticle patterned array. The shapes at an ion beam of 30° was similar to those at 20° but sharper shapes was observed. Figure 5 (e) and (f) shows SEM images at an ion beam exposure angle of 60° with respect to the normal direction of the substrate with a square type nanoparticle patterned array. The images showed a distinctively tilted mushroom-like-shape etched appropriately by the ion beam. Figure 5 (g) and (h) present SEM images at an ion beam exposure angle of 70° with respect to the normal direction of the substrate with a square type nanoparticle patterned array. The images showed a more distinctively tilted mushroom-like-shape that might be useful for creating new functions of nano-circuits. More non-spherical forms that are useful for a range of applications can be obtained by controlling the exposure time, exposure energy and exposure angle.

## Conclusions

This paper proposed a practical technique for making a non-spherical nanoparticle array by colloidal etching without any mask through low energy ion beam irradiation. In this experiment, the spherical nanoparticle array was transferred by a soft nanolithography technique, which is a simple and effective pattern transfer method for nanostructures on the surface of a thin adhesive polymer on a plane substrate, after placing spherical nanoparticles on a patterned PDMS [poly(dimethysiloxane)] stamp produced from a patterned Si wafer using a rubbing technique. The resulting non-spherical nanoparticle array was modified from a spherical nanoparticle array by ion beam irradiation. A well-arrayed layer of cone-like-shapes was produced at various exposure times under a head-on ion beam exposure. In addition, a variety of mushroom-like-shapes were produced on a substrate with a well-arranged spherical nanoparticle array depending on the exposure angle.

This technique is a fast and simple method for fabricating reproducible and controllable patterned nanoparticle arrays, and is expected to have potential applications in the nanophotonics, field emission displays (FED), microfluid devices, and bio sensor and particularly for light extraction in organic light emitting diode (OLED), gas detector with high sensitivity, improving light efficiency of solar cell^19^,^20^,^21^,^22^,^23^,^24^,^25^,^26^.

## Methods

### Sample preparation of patterned Si wafers

The Si-wafers patterned with square, rhombic and hexagonal arrays of protrusion in the Korea Nanofab Center positioned in Suwon, Korea were fabricated by using a positive photoresists (PR). The PR (DHK-2323) was purchased from Dongjin Semichem Company and used without further purification. An anti-reflection (AR) layer (DARC K131, Dongjin Semichem) was spin-coated on a Si-wafer at 4000 rpm during 50 s. The AR/Si wafer was baked in an oven at 200°C for 1 min. After a positive PR was spin-coated onto the AR/Si wafer at 4000 rpm for 50 s, the PR/AR/Si wafer was baked at 110°C for 1 min. The patterns needed were designed with stepper photomask (SMIC's MASK Co.) The PR/AR/Si wafer to fabricate nanopatterns desired was exposed by the KrF UV light (λ = 248 nm under a patterned stepper photomask. The irradiation energy depends on the dimension of size of protrusion in the wafer. Here, the exposure energy to fabricate protrusion of diameter 600 nm and the pitch (600, 900, and 1100 nm) was exposed to the PR/AR/Si wafer with 100 mJ/*cm^2^*. The PR/AR/Si wafer was developed by using a AZ 300 MF developer (Clarient Co.). By ion coupled plasma (ICP) using *SF*_6_ with deep etching of the PR-removed Si-wafer regions, the high patterned Si-wafer was fabricated. The etching was carried out until the etching depths became the needed value (350 nm). The remaining PR and AR layers after etching were removed by a PR asher. The size of a patterned Si wafer was 0.5 × 0.5 cm^2^.

### Transfer and shape-modifaction of a nanopattened arrays

The patterned Si-wafers were cleaned ultrasonically with toluene for 5 min and washed with deionized water. After drying the Si-wafers under flowing nitrogen, the wafers were placed into a petri-dish. The mixture of the PDMS which was mixed by 90 wt% pre-polymer (Sylgard 184A) and 10 wt% curing agent (Sylgard 184B) was poured onto a petri-dish, which was placed by patterned Si-wafers on the bottom. The mixture was kept in air for 30 min at 25°C to remove gas bubbles from the liquid PDMS-mixture. The mixture included the wafer was then cured in an oven for 1 h at 80°C. After curing, it was peeled off from the patterned Si-wafer, and cured in an oven for 1 h and 30 min at 100°C to further harden the patterned PDMS stamp. After a small amount of nano silica particles (2 ~ 3 mg) was placed on the patterned PDMS stamp, the nanoparticle powder was rubbed smoothly using a flesh PDMS [poly(dimethysiloxane)] plate (3.0 × 3.0 × 0.25 *cm*^3^) to a unidirection repeatedly for roughly 30 s and we checked whether the surface of the PDMS stamp was covered with nanoparticles or not by using dimly iridescent on reflection of light. Generally, after the above procedure, monolayers of silica nanoparticles assembled on a patterned PDMS stamp by rubbing are usually contaminated with a small amount of the aggregated nanoparticles. The randomly aggregated upper nanoparticles layers were removed from the patterned nanoparticle array on the patterned PDMS stamp by slightly pressing a fresh PDMS plate onto the nanoparticles layers on the PDMS stamp followed by removing the PDMS plate repeatedly, which could be leaved only high patterned first layer array of nanoparticles that is more strongly bounded to the patterned stamp by subsequently removing the PDMS plate. To transfer the pattern nanoparticles array onto a glass substrate, a glass substrate was spin-coated with the UV curable polymer (NOA60, Norland Products Inc.) solvent at 4000 rpm for 75 s.

The concentration of the NOA60 polymer in toluene was 7 wt.%, which corresponds to a film thickness of approximately 30 nm after the spin coating. The patterned nano silica particle array placed on the PDMS stamp was placed in conformal contact on a NOA60-coated glass substrate. After exposure to UV light (λ = 365 nm) for 20 min to anchor the nanoparticles to the glass substrate, the PDMS stamp was peeled off from the NOA60-coated glass substrate, and the array was transferred to a glass substrate with the same formation of mirror symmetry. The patterned nano silica particle array transferred to the substrate was annealed at above 500°C for 2 h to remove the NOA60 polymer film coated on a substrate. The size of the nanoparticle array transferred on the substrate was 0.5 × 0.5 cm^2^. We could obtain almost uniformly covered nanoparticles array at 17 out of 56 tested samples of size of 25 cm^2^. In our experiment, thus, yield was about 30%. The point defects of the patterned nanoparticles layer in the range were observed with less than ten of the particles.

**Figure 6:**
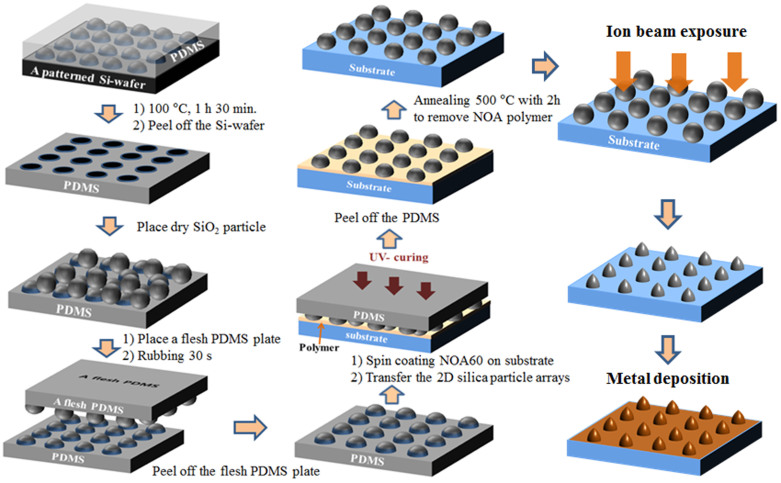
Schematic illustration of the fabrication process for obtaining a patterned monolayer array on a substrate of silica nanoparticles and a metal deposition on the modified surface.

Figure 6 presents a schematic diagram of the entire fabrication process to obtain a patterned monolayer array of silica nanoparticles on the substrate. The patterned colloidal particle array on the glass substrate after removing the polymer layer was bombarded with a low-energy Ar ion beam. As an ion source, a cold hollow cathode (CHC) is used to yield a linear type ion beam. In order to collimate the ion beam, two perforated grids are used as elecro-focusing lenses. The CHC represents a separate cooled chamber, which is equipped with a magnetic system, and is connected to a discharge chamber through an orifice. Argon gas feeding into the ion source is carried out through the CHC only. Discharge ignition in the cathode takes place at nominal discharge voltages and nominal gas flow rates. A neutralizer filament located outside of the ion source serves as a source of electrons necessary for the compensation of ion beam spatial charge and reduces the repulsive force among ions. The working and base pressures of the ion beam system were 2 × 10^−4^ Torr and 3 × 10^−6^ Torr based on flowing Ar gas, respectively. The ion beam energy and beam current density were fitted with 600 eV and 500 μA/cm^2^, respectively. The exposure time and exposure angle according to the samples were changed from 2 min to 4 min and 30 s at a head-on irradiation and 20°~70° with an exposure time of 4 min and 30 s, respectively, to assess the dependence of the shape of the spherical colloidal particle on the irradiated time and angle (Supporting information Figure S3). A layer of Al (40 nm) was deposited on the patterned nanoparticle arrays on a substrate irradiated with a low-energy Ar ion beam for 10 minutes by RF magnetron sputtering. Deposition was performed in 20 sccm of flowing Ar under mass flow-meter control. The working and base pressures of the sputtering system were 8 × 10^−4^ Torr and 3 × 10^−6^ Torr, respectively.

## Author Contributions

J.S.G. planned the experiment; K.C.H. performed the experiments. All authors analyzed the data and wrote the manuscript.

## Supplementary Material

Supplementary Information Supplementary information
