# Supplementary material for: Shape-modification of patterned nanoparticles by an ion beam treatment
Source: Sci Rep. 2015 Feb 17;5:8523. doi: 10.1038/srep08523 (PMC4330533; doi:10.1038/srep08523)
Supplement: Supplementary Information [file srep08523-s1.pdf]

Supplementary information:

Shape-modification of patterned nanoparticles by an ion beam treatment

Kyong Chan Heo and Jin Seog Gwag*

Department of Physics Yeungnam University, 214-1 Dae-dong, Gyeongsan 712-749, Korea

*Corresponding Author: sweat3000@ynu.ac.kr

Experimental Methods

We also obtained a similar result of transferring non-closed silica particle arrays onto a planar glass substrate using the soft lithography method with polyethyleneimine (PEI) as molecular glue.

Figure S1 shows the SEM image of a patterned Si-wafer used to fabricate a nanopatterned PDMS stamps. The Si wafer was patterned with a cubic array of cylindrical shape. The top diameter, bottom diameter and the high of each cylinder are roughly 600, 700 and 350 nm, respectively. The center-to-center distance between the nearest silica cylinders is approximately 1.1 μm.

Figure S2 exhibits a process for the transfer on a glass substrate of patterned nanoparticles arrays using the soft lithography method. For transferring the patterned nanoparticle array onto a glass substrate, a glass substrate was spin-coated by using polyethyleneimine (PEI) solvent at 4000 rpm for 75 s instead of NOA polymer as molecular glue. The concentration of PEI in toluene was 3 ~ 4 wt.%. After the sample was heated at 100 °C for 2 h, the PDMS stamp was carefully peeled off, leaving patterned nanoparticle array on the PEI-coated glass substrate. The patterned nano particles array transferred to the substrate was baked at higher 500 °C with 1.5 h to eliminate the PEI polymer on substrate

Figure S3 shows schematic illustration of controlling shape-modification of patterned nanoparticle arrays by ion beam treatment and a metal deposition on the modified surface.

Results and discussion

Figure S4 shows a large area scanning electron microscopy (SEM) image of the patterned nanoparticles arrays annealed in air at 500 °C for 2 h to remove the NOA60 polymer film on the glass substrate. This result shows a large area of well-arranged 2D nanoparticle arrays ordered on a planer glass substrate. The ordered nanoparticles on the substrate were stable without aggregation of the patterned nanoparticle array by 550 °C. We could obtain almost uniformly covered nanoparticles array in 17 out of 56 tested samples of size of 25 mm^2. In our experiment, thus, yield was about 30 %.

Figure S5 shows a large area SEM image of Al-deposited patterned nano silica particle array on the substrate prepared under the ion beam exposure at 600 eV for 4 min and 30 s from normal to the surface direction. The bottom inset shows a higher-magnification of its image. Furthermore, the SEM image also revealed that the distance between the nearest arrays built up by ion beam exposure retained roughly their center-to-center distance without being dissipated or blown away under the Al-deposition. Furthermore, when a spherical nanoparticle arrays was modified to another non-spherical nanoparticle arrays by ion beam exposure, controlling the morphology of nanoparticle arrays patterned with NOA polymer was more stable than it with PEI. In addition, in case of using the NOA polymer as a molecular, the organization process is simpler and faster than it using PEI glue.

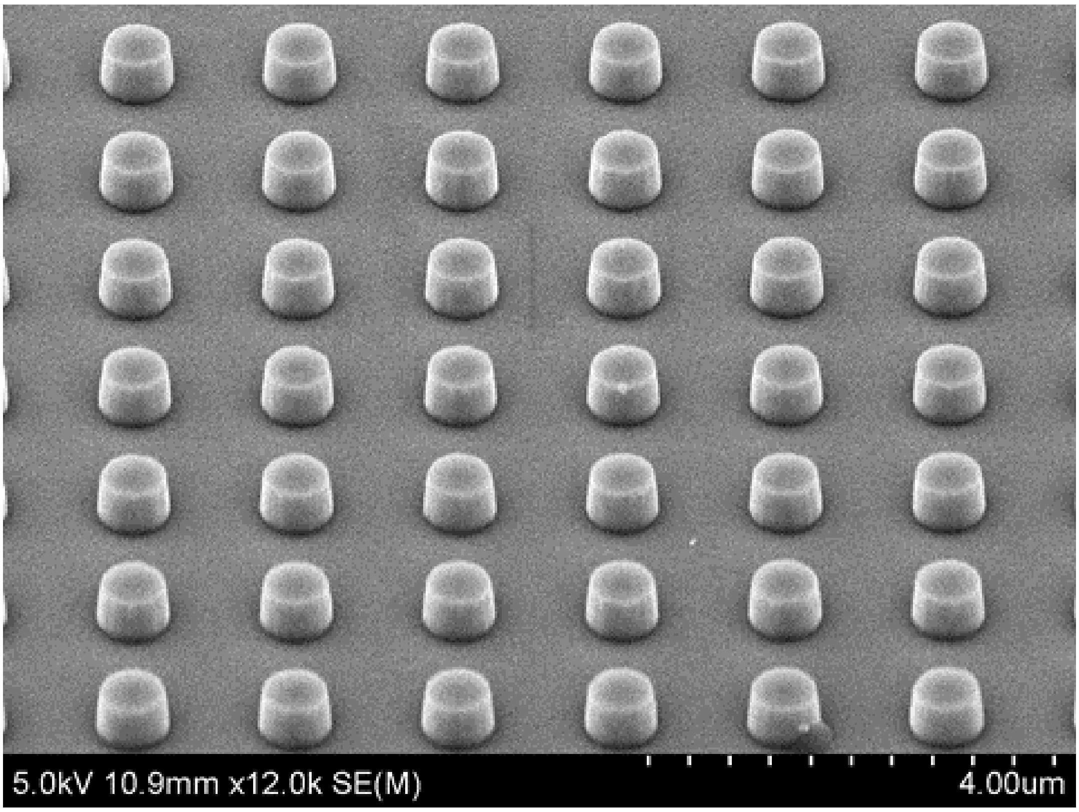

Figure S**1**. **SEM images of a patterned Si-wafer used to obtain a nanopatterned PDMS stamp.**

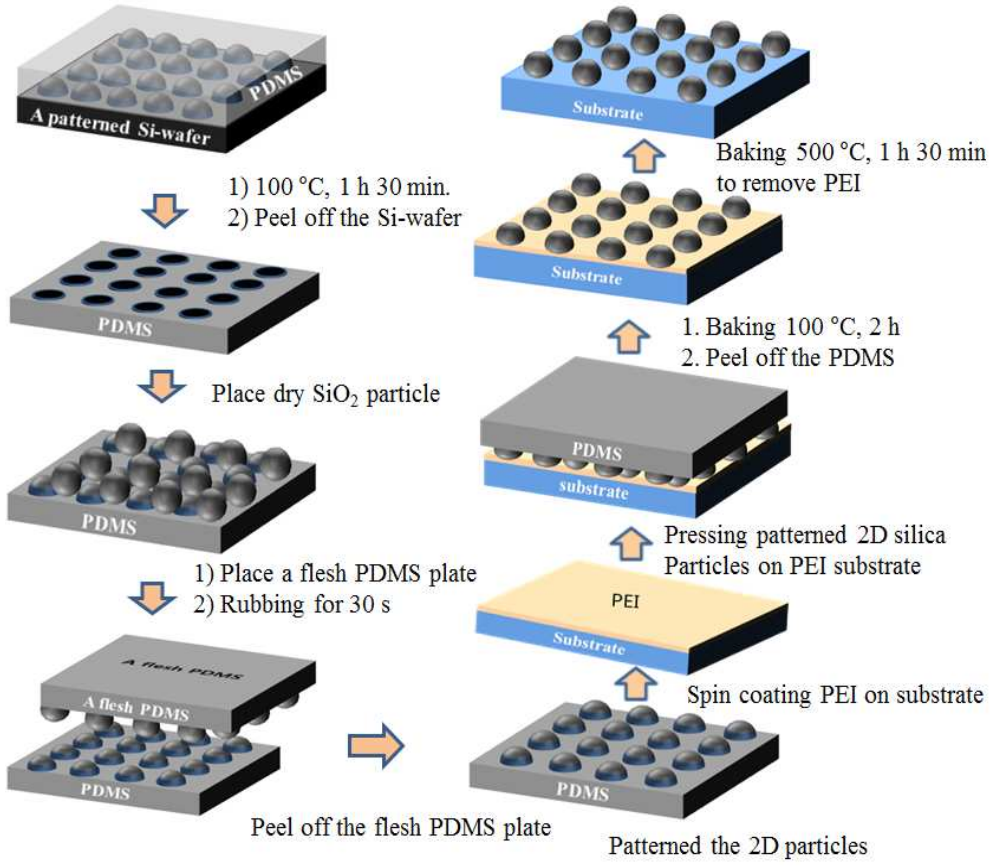

Figure S2. **Schematic illustration of the procedure to prepare patterned monolayer arrays on a substrate of silica nanoparticles.**

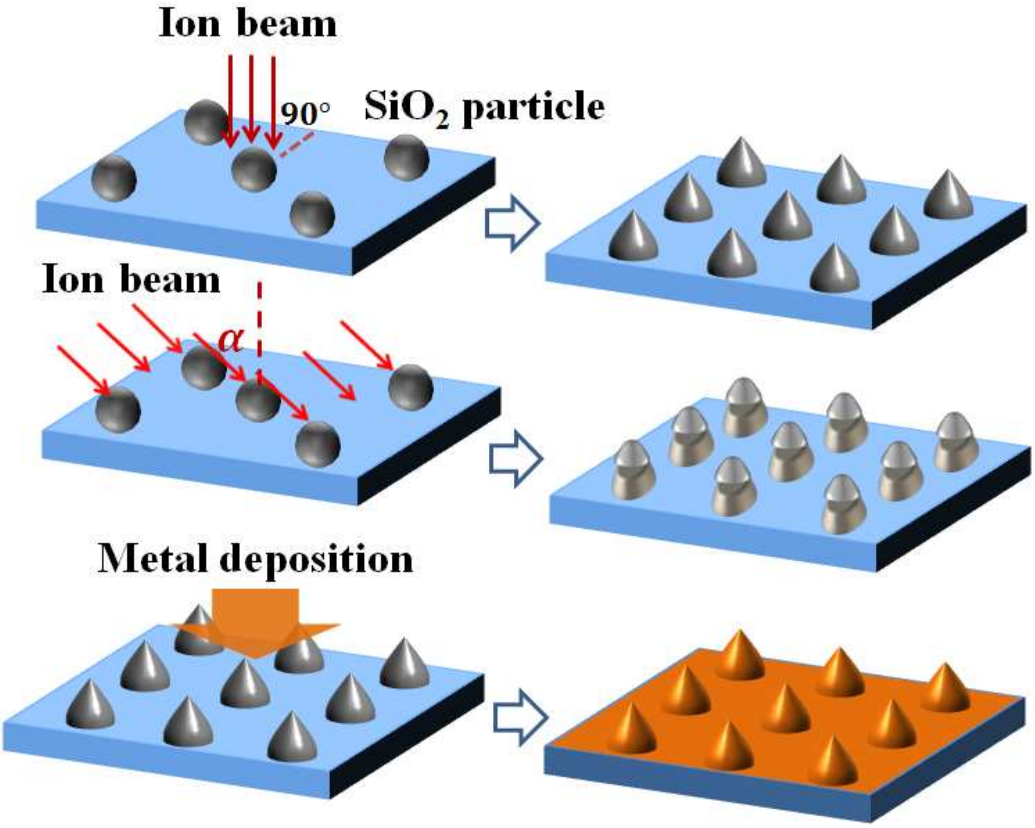

Figure S3. **Schematic illustration of controlling shape-modification of patterned monolayer arrays by ion beam treatment and a metal deposition on the modified surface.**

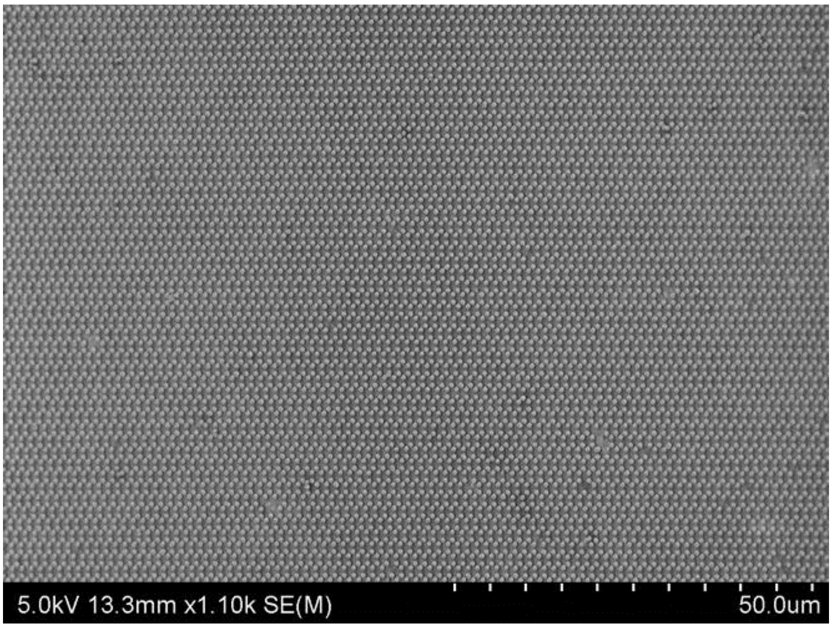

Figure S4. SEM image of the morphology of silica particles arranged in the hexagonal annealed in air at 500 °C for 2 h to remove the NOA60 polymer film on the substrate.

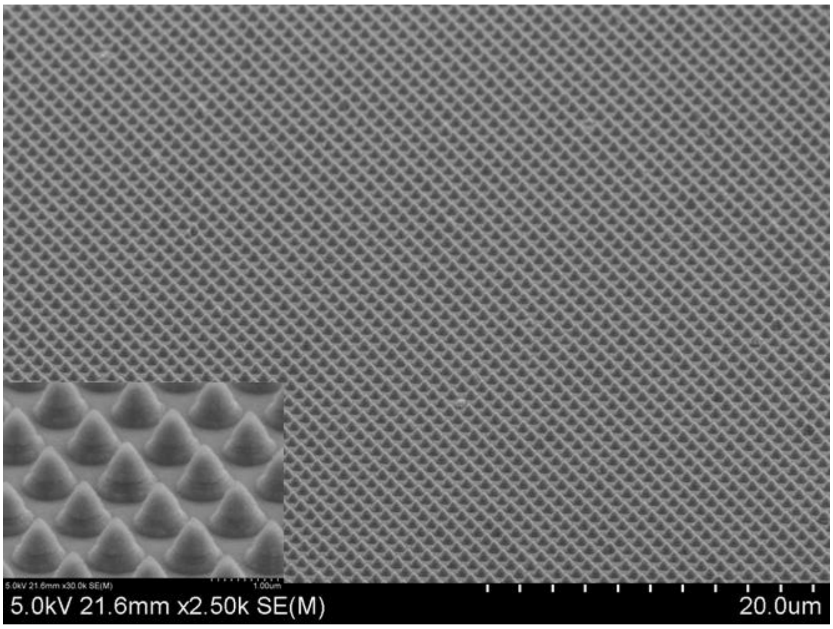

Figure S5. SEM image of Al-deposited patterned colloidal silica particle array on the substrate prepared under the ion beam exposure at 600 eV for 4 min and 30 s. The bottom inset showing a higher-magnification image.
